# Considerations of Control Conditions Designs in Randomized Controlled Trials of Exercise Interventions for Cancer Survivors

**DOI:** 10.1177/08445621211062467

**Published:** 2022-02-04

**Authors:** Wing Lam Tock, Christine Maheu, Nathalie A. Johnson

**Affiliations:** 1 Faculty of Medicine and Health Sciences, Ingram School of Nursing, 5620 McGill University, Montréal, Canada; 2 Department of Medicine, Division of Experimental Medicine, Faculty of Medicine and Health Sciences, 5620 McGill University, Montréal, Canada

**Keywords:** randomized controlled trials, methodology, control conditions, study design, exercise interventions

## Abstract

**Background:**

Given the multifaceted complexity in the nature of randomized controlled trials, identifying an appropriate and comparable control condition is an essential step to ensure methodological rigor, which allows for researchers to draw unambiguous conclusions concerning the efficacy of the intervention being studied.

**Objectives:**

The objectives of this paper are to (a) review the current literature and analyze the control condition designs in exercise interventions targeted for cancer survivors; (b) provide an overview of the benefits and limitations of various types of control conditions used in exercise interventions; (c) discuss the considerations in the design of control conditions for exercise interventions; and (d) suggest recommendations for control condition design in future trials of behavioral interventions.

**Results:**

The review of randomized controlled trials of exercise training interventions for cancer survivors revealed that the design of control conditions varied. The most commonly employed design could be classified into two major categories: (a) active controls including attention control, add-on controls, and dismantling controls; and (b) inactive controls including no-treatment, usual care, and wait-list control. Examples from the literature are presented. Four principal considerations concerning control condition design, including appropriateness, credibility, appeal, and comparability, are discussed. Recommendations on how to avoid some major threats to validity and potential biases are also provided.

**Conclusions:**

Careful planning for the control group design is as important as for the intervention group. Researchers can use the considerations presented in the paper to assist in planning for the most appropriate control condition for their study.

## Background and Objectives

### Background: Exercise Intervention for Cancer Survivors

Advances in cancer treatment and early detection efforts have yield encouraging results. The 5-year survival rate for all types of cancer has improved markedly in Canada since the early 90s (Canadian Cancer Society, 2020). The growing number of cancer survivors in the world has warranted the development of targeted health behavioral health interventions to mitigate the adverse effects of cancer diagnosis and the toxicities of treatments (Tollosa et al., 2019). While researchers have discovered a myriad of behavioral interventions that can benefit the health of cancer survivors, exercise training remains one of the options with the highest therapeutic value on both psychological and physical health (e.g., improved physical functioning and quality of life, and mitigation of anxiety) for this patient population (Liu et al., 2019; Segal et al., 2017).

The World Health Organization recommends that adults (aged 18- 64) should engage in a minimum of 150 min of moderate to vigorous-intensity aerobic physical activity (MVPA) per week (World Health Organization, 2020). While these guidelines were initially extended to cancer survivors, experts in the field have since reviewed the literature and recently published a series of evidence-based exercise prescriptions specifying the frequency, intensity, time, and type of exercise training for cancer survivors during and post-chemotherapy (Campbell et al., 2019). Besides the conventional aerobic or resistive exercise regimes, interventions using a variety of exercise modalities have also shown effectiveness in improving overall health status among cancer survivors (Stout et al., 2017). For the purpose of this study, exercise intervention is categorized as a sub-type of behavior interventions, of which the content comprises training that involves bodily movement aiming at increasing energy expenditure. Exercise training comprises structured, repetitive, and purposeful activities that gear towards enhancing or preserving one or more components of physical health (e.g., cardiorespiratory endurance, balance, flexibility, and musculoskeletal strength) (Campbell et al., 2019; Caspersen et al., 1985; Wolin et al., 2012).

### Randomized Controlled Trials of Exercise Interventions

Exercise interventions are often considered complex interventions because they typically comprise multiple components. For instance, interventionists might be required to perform a variety of complex behaviors while delivering the treatment, the intervention design might consist of personalized and tailored activities, there might be multiple levels of study outcomes, and a large number of stakeholders can be involved at different stages of the process (Hecksteden et al., 2018; O’Cathain et al., 2019). For complex behavioral intervention studies, the phase III evaluation stage predominately relies on Randomized Controlled Trials (RCTs) as the gold standard experiment design in estimating the efficacy of the intervention, as well as the causal relationships in the study design (Hecksteden et al., 2018; Polit & Beck, 2021; Sidani, 2015). With increasing evidence supporting the relationship between exercise and physical functioning and psychological health among cancer survivors, clinical trials investigating the effectiveness of exercise intervention have been proliferated in the realm of cancer survivorship care in the past decades (Segal et al., 2017; Zhi et al., 2019). In fact, given that the methodological approach to conduct exercise training interventions is multifaceted and complex, an extension checklist of the CONsolidated Standards Of Reporting Trials (CONSORT) Statement titled the Consensus on Exercise Reporting Template (CERT) had been published in 2016 in order to provide supplementary information to report and document RCTs of exercise interventions (Slade et al., 2016).

The RCT is a robust study design most frequently used in pharmacological trials to establish safety, therapeutic efficacy, and tolerability of newly developed drugs or therapies. The gold standard design of an RCT consists of a double-blinding procedure, and placebo control, which is a pharmacologically inactive agent used to evaluate the effects of being given medications in the experimental group (U.S. Department of Health and Human Services, 2020). Choosing a proper control condition is one of the most fundamental yet critical aspects of the design in an RCT. The control is an essential element that allows the researcher to discriminate whether a treatment has an effect that is due to a hypothesized mechanism of action, and is not attributable to nonspecific effects or other confounders (e.g., sources of bias) (Sidani, 2015). In behavioral intervention studies, researchers can infer the improvements among participants receiving the intervention treatment to the salient features of the intervention itself based on the results of a well-controlled study.

### Methodological Issues

Many nonspecific factors such as therapeutic environment, social interaction, and attention from research staff can impact the outcome of a RCT. A properly designed RCT of behavior intervention should allow for the identification of the “active ingredients” responsible for the desired behavior (Edmond et al., 2019; Sidani, 2015). To achieve this, the control condition should not have a significant effect on the hypothesized mechanism of action that explains the effectiveness of the intervention. Unfortunately, the active and inactive components in the intervention treatment are not as well defined as those of medications in pharmacological trials, given that behavioral interventions are often complex (O’Cathain et al., 2019). Further, since behavior intervention RCTs often rely on interpersonal interactions between participants and clinicians, blinding is challenging given everyone is usually aware of the true nature of their treatment allocation. Consequently, the gold standard design for RCTs is less clear in studies investigating the effectiveness of behavioral interventions (Hecksteden et al., 2018).

In exercise science, RCTs adopting robust methodologies are required to provide valid scientific evidence on the types of exercise, training routine, and dosage appropriate for different populations (Hecksteden et al., 2018). Yet, methodological consistency of RCTs of exercise intervention targeting cancer survivors has not been established, particularly in the choice of comparable control conditions (Boutron et al., 2017; Hecksteden et al., 2018; Pinto & Floyd, 2007). Since it is not always possible to develop a deceptive/mimic intervention (i.e., the equivalent to a placebo medication) that generates solely a placebo effect in RCTs of behavioral interventions, researchers have several options for their choice of comparison conditions including inactive control (i.e., wait-list control and usual care) and active/attention control (i.e., in which the participants engage in some activities or tasks during the intervention period.) (Segal et al., 2017; Zhi et al., 2019).

### Inactive and Active/Attention Controls

Using an inactive control is a common practice in RCTs of behavioral interventions due to the challenge of identifying an appropriate behavioral “placebo”. Inactive conditions allow for the researchers to detect the outcome of the experimental intervention as compared to that of an un-intervened control group (Karlsson & Bergmark, 2015; Street & Luoma, 2002). The terminologies used to describe the subtypes of inactive controls have not yet been standardized, but they generally include usual care (standard care), no treatment, and wait-list control (Locher et al., 2018; Street & Luoma, 2002). In RCTs adopting usual care as the comparator, participants in the control arm receive the care they would normally get for their clinical conditions. Depending on the nature of the study, the research interventionists might also recommend that the participants maintain their usual habits or activity level, which applies to the context of an exercise training study (Lindquist et al., 2007). The terminology “no treatment” is less common practice in behavioral intervention RCTs; generally, the control arm not performing the intervention activities is described as usual care. Finally, in a wait-list controlled study, participants who are randomized to the control arm receive the intervention treatment upon the completion of the designated study time frame. Participants in the wait-list control arm typically receive usual care during the delay (Elliott & Brown, 2002; Lindquist et al., 2007; Street & Luoma, 2002).

Active controls are sometimes used in RCTs to account for the nonspecific effects of the intervention in a similar way that placebo medications are used to control for expectancy effects in a pharmaceutical trial (Aycock et al., 2018). Active controls are generally recognized as attention controls in behavioral intervention studies, given that participants receive an activity that is an inactive substitute of the intervention, with a similar amount of attention and contact (Aycock et al., 2018; Lindquist et al., 2007; Street & Luoma, 2002). Sub-types of attention controls include several types of component controls, where components of a pre-established intervention package are added on (i.e., additive control), or isolated from (i.e., dismantling control) the study arms in an attempt to identify the active ingredient hypothesized to contribute to the desired changes (Lindquist et al., 2007). In RCTs with multiple study arms, the experimental group is compared to two control groups, with one being a usual care/no-treatment group, and the other control group being an active attention control.

### Objectives

Given the multifaceted complexity in the nature of RCTs of exercise intervention and the specialized needs of cancer survivors, identifying an appropriate and comparable control condition is an essential step to ensure methodological rigor, which allows for researchers to draw unambiguous conclusions concerning the efficacy of the exercise training treatment being studied (Hecksteden et al., 2018; Karlsson & Bergmark, 2015). Because the design of control condition continues to be a challenge for researchers, the objectives of this paper were to (a) review the current literature and analyze the control condition designs in exercise interventions targeted for cancer survivors; (b) provide an overview of the benefits and limitations of various types of control conditions available for exercise intervention studies; (c) provide perspectives on considerations in the design of control conditions for exercise interventions; and (d) suggest recommendations for control condition design in future exercise intervention RCTs in cancer survivorship research. Given the authors’ specialization in oncology research, the literature reviewed in this paper are focused on the cancer survivorship studies. The motivation for writing this paper arose due to the challenges faced by the authors of this paper while undertaking an exercise intervention studies for cancer survivors. It is hoped that the cancer survivorship literature can elucidate some of the more salient issues in control condition design. While this paper primarily focuses on exercise training intervention adapted to cancer survivors, the issues and dilemmas explored here are applicable to other behavioral intervention research commonly conducted by nursing researchers such as diet modification, symptom self-management, and mindfulness intervention, etc.

## Methods and Procedures

To examine the control conditions designs in RCTs evaluating the effects of exercise interventions for cancer survivors in current literature, a comprehensive literature search was conducted. Three databases, CINAHL, Medline_Ovid, and EMBASE, were searched using Boolean search strategies with truncated keywords. The search strategies for CINAHL are shown in Table 1. Criteria for inclusion were as follow: (1) RCTs of any exercise training interventions involving different training modes, such as aerobic, resistance, weight, and flexibility training were included; (2) the exercise training interventions could be conducted in different settings including home-based or community-based studies; and (3) participants involved in the RCTs had a confirmed diagnosis of any type of cancer, and had received and completed curative treatment. Studies included in the analysis were published in English since 2015.

**Table 1.** Search Strategy for CINAHL.

S1 (MH “Cancer Survivors”)
S2 “cancer survivor*”
S3 “exercise therapy”
S4 (MH “Physical Activity”)
S5 (MH “Physical Fitness”)
S6 (MH “Nursing Interventions”)
S7 (MH “Therapeutic Exercise”)
S8 “exercise program”
S9 (MH “Randomized Controlled Trials”)
S10 S3 OR S4 OR S5 OR S6 OR S7 OR S8
S11 S1 OR S2
S12 S9 AND S10 AND S11
Limit applied
Published Date: 20150101-20210801

Given the present review's focus on exercise training interventions, studies were excluded if the intervention involved mixed components combining exercise with other therapeutic approaches, such as psychotherapy, dietary modification, or cognitive-behavioral counseling. Finally, trials that compared exercise training with pharmacological and surgical treatments were excluded. When multiple publications from a single RCT were found, only the primary publication was included for this analysis in order to avoid double counting of studies using the same trial design.

## Results

### Findings

**Figure 1.**
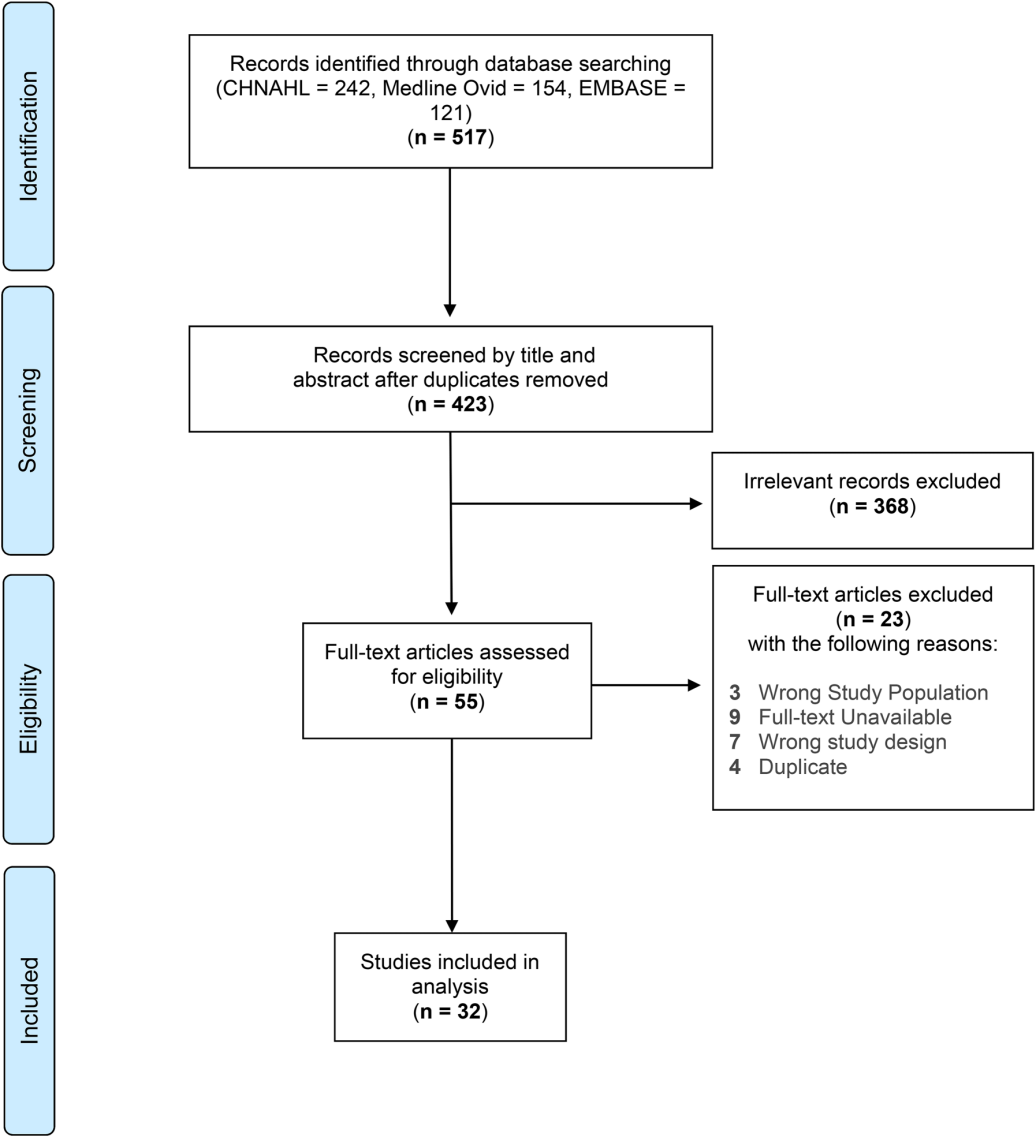
Study selection flow diagram.

After screening procedures, 32 articles were selected for inclusion in this analysis (Figure 1). The review of RCTs of exercise training interventions for cancer survivors revealed that the design of control conditions varied. The most commonly employed design could be classified into two major categories: (a) inactive controls including no-treatment, usual care, and wait-list control; and (b) active controls including attention control, add-on controls, and dismantling controls, as shown in Table 2**.** A total of 25 of the 32 studies (78.1%) meeting the search criteria employed inactive control condition design, while seven out of the 32 studies (22.6%) employed an active control condition design. Among the 32 included studies, 5 studies had more than two arms in their design, all of which consisted of one arm receiving usual care as the control. Finally, the majority of the RCTs were conducted among breast and colorectal cancer survivors (n = 25, 78.1%). The following section provides a detailed description of the various types of control condition designs used in these studies.

**Table 2.** Comparison of Control and Intervention Group Components in Exercise RCTs for Cancer Survivors.

Authors	Year	Target population (N, C, I) *	Control condition	Intervention treatment(s)
*Active Control (n* *=* *7)*				
Brown, & Schmitz	2015	Female breast cancer survivors (N = 294, C = 146, I = 148)	Maintain baseline level of physical activity; 13 weeks of supervised exercise instruction; 12 months membership to a community fitness center	Weightlifting program for 12-months; 13 weeks supervised weightlifting instructions; 12 months membership to a community fitness center
Devin et al.	2016	Colorectal cancer survivors (N = 47, C = 17, I = 30)	4 weeks of moderate-intensity exercise (MIE)	High intensity exercise (HIE) training
Knobf et al.	2016	Female breast, gynecologic or colorectal cancers or lymphoma survivors (N = 154, C = 78, I = 76)	Home-based health promotion program based on national guidelines for 30 min of moderate intensity activity most days of the week	Supervised 12-month aerobic-resistance exercise intervention at a community fitness center for 3 times per week
Pinto et al.	2015	Breast cancer survivors (N = 76, C = 37, I = 39)	Contact control: 12-week American Cancer Society's Reach to Recovery (RTR) program	12-week RTR program + recommendations of 30 min of more of moderate intensity PA on most days of the week
Schmitt et al.	2016	Breast cancer survivors (N = 28, C = 14, I = 14)	A 3-week low-to-moderate intensity exercise program	A 3-week multimodal rehabilitation program involving high intensity interval training program
Schwartz et al.	2015	Cancer survivors (N = 50, C = 25, I = 25)	A 12-week supervised 1-on-1 exercise program (Cancer Fitness Fundamentals program)	The 12-week Cancer Fitness Fundamentals exercise program + Restwise (Recovery Science & Technology, LLC; Con- cord, MA) which is an online recovery assessment tool
Zhou et al.	2017	Ovarian cancer survivors (N = 144, C = 70, I = 74)	Attention control included weekly phone calls from a staff member, along with a 26-chapter book that only contained ovarian cancer survivorship–related information	A 6-month home-based exercise program targeted at 150 min per week of moderate intensity aerobic exercise per week facilitated by weekly telephone calls
*Studies with Multiple Arms (With the control condition being an inactive design) (n* *=* *5)*				
Brown et al.	2018	Colon cancer survivors (N = 39, C = 13, Ia = 14, Ib = 12)	Usual care	a) 150 min per week of aerobic exercise (low-dose) for 6 months b) 300 min per week of aerobic exercise (high-dose) for 6 months
García-Soidán et al.	2020	Breast cancer survivors (N = 316, C = 79, Ia = 79, Ib = 79. Ic = 79)	The control group should not make any changes in their lifestyle, incorporating any new physical activity	2 years duration a) Strength training program b) Aqua fitness program c) Aerobic exercise program
Kampshoff et al.	2015	Breast, colon, ovarian, cervix or testis cancer, or lymphomas survivors (N = 277, C = 91, Ia = 91, Ib = 95)	Wait list control	a) 12-week high intensity (HI) resistance exercise program b) 12-week low-to-moderate intensity (LMI) resistance and endurance exercise program
Nouri et al.	2020	Breast cancer survivors (N = 75, C = 25, Ia = 25, Ib = 25)	Usual care	a) Resistance training group b) Combined training group (resistance + core stability training)
Park et al.	2015	Colorectal and breast cancer survivors (N = 162, C = 59, Ia = 53, Ib = 50)	Conventional treatment consultation (usual care)	a) Oncologist's exercise recommendation combined with an exercise motivation package b) Oncologist's exercise recommendation (exercise: at least 150 min of moderate level physical activity and strengthening exercise twice a week.)
*Inactive Control (n* *=* *20)*				
Arem et al.	2016	Female breast cancer survivors (N = 121, C = 60, I = 61)	Usual care	150 min per week of moderate-intensity aerobic exercise and twice-weekly supervised strength training
Cantarero-Villanueva et al.	2016	Colon cancer survivors (N = 46, C = 23, I = 23)	Usual care: general recommendations for a healthy lifestyle that were delivered at the start of the program in paper forma	An 8-week trunk muscle stabilization exercise program group (CO-CUIDATE) for 3 times per week
Chang et al.	2020	Breast cancer survivors (N = 46, C = 23, I = 23)	Wait-list control: Instructed to maintain their routine occupational and leisure-time physical activity during the study period	A 12-week program with combined aerobic and resistance exercise regimen
Craike et al.	2018	Prostate cancer survivors (N = 147, C = 93, I = 54)	Usual care which typically does not include recommendations for PA	The 12-week exercise program with 150 min per week of home-based, supervised moderate–vigorous PA
Dhillon et al.	2017	Lung cancer survivors (N = 112, C = 55, I = 56)	Usual care with general health education materials only	An 8-week physical activity program plus general health education materials
Dieli-Conwright et al.	2018	Breast cancer survivors (N = 100, C = 50, I = 50)	Usual care (to maintain their current level of physical activity)	A 16-week program: exercise program with 150 min of aerobic exercise and 2 to 3 days of resistance exercise training per week
Ebrahimpour et al.	2021	Breast cancer survivors (N = 30, C = 15, I = 15)	Usual care (participants are advised to perform their routine daily activities)	A 12-week program of concurrent yoga and Pilates training, 3 sessions per week and 75 min each
Galiano-Castillo et al.	2016	Breast cancer survivors (N = 81, C = 41, I = 40)	Usual care with basic recommendations in written format for exercise	An 8-week Internet-based, tailored exercise program
Hagstrom et al.	2016	Breast cancer survivors (N = 39, C = 19, I = 20)	Usual care	Resistance training 3 times per week for 16 weeks, sessions lasted 60 min each
Hartman et al.	2017	Breast cancer survivors (N = 87, C = 44, I = 43)	Waitlist wellness-contact control (via emails)	Gradually increasing aerobic exercise over time to target at least 150 min of MVPA per week
Kim et al.	2019	Colorectal cancer survivors (N = 71, C = 34, I = 37)	Usual care: participants were instructed to continue with their usual activities or exercises during the intervention	A 12-week home-based exercise program aimed to increase the level of PA to 18 ∼ 27 metabolic equivalent of task (MET) hours per week
Lee et al.	2017	Colorectal cancer survivors (N = 123, C = 61, I = 62)	Standard care	A 12-week home-based exercise consisting of aerobic and resistance training, with a goal of obtaining ≥18 metabolic equivalent task (MET) per week
Manchola-González et al.	2019	Acute Lymphoblastic Leukemia survivors (Children and adolescents) (N = 24, C = 121, I = 12)	Usual care: participants were advised to continue their usual activities with no restriction placed on physical activity throughout the study period	A 16-week program involving a 90-min home visit by a trained physiotherapist, and strength, flexibility, and aerobic exercises 3 days per week
Mardani et al.	2021	Prostate cancer survivors (N = 80, C = 40, I = 40)	Routine healthcare for the treatment of prostate cancer and instructions to maintain their customary physical activities and dietary patterns	12-week exercise program including aerobic, resistance, flexibility, and pelvic floor muscle exercises
Rogers et al.	2017	Breast cancer survivors (N = 222, C = 112, I = 110)	Usual care included in the printed materials from the American Cancer Society	A 3-month social cognitive theory-based program including 12 supervised exercise sessions with an exercise specialist. The exercise sessions were tapered over the first 6 weeks to an exclusively unsupervised home-based program
Santos et al.	2019	Breast cancer survivors (N = 25, C = 13, I = 12)	Requested not to change their physical activity habits	Highly supervised resistance training program (1:1 coach to patient ratio), once per week for 8 weeks
Scruggs et al.	2018	Breast cancer survivors (N = 60, C = 25, I = 35)	Standard care	24-week, group-based program including group sessions of 90 min each
Tabatabai et al.	2019	Breast cancer survivors (N = 206, C = 103, I = 103)	Usual care receiving a monthly health newsletter	12-month exercise program with a combination of resistance training and aerobic exercise administered through the Young Men's Christian Association
Winters-Stone et al.	2018	Breast cancer survivors (N = 95, C = 45, I = 50)	Usual care with an oncologist verbal recommendation to exercise	An oncologist verbal recommendation to exercise plus a cancer-specific yoga DVD, for a low-intensity and restorative 30-min exercise program
Ying et al.	2019	Breast cancer survivors (N = 86, C = 40, I = 46)	Usual care and requested to maintain their original physical activity	Baduanjin exercise 3 days/week at hospital and another 4 days/week at home for 6 months

*Note: N = total number of participants in the trial; C = number of participants in the control arm; I = number of participants in the intervention arm, if more than one intervention arms were used, Ia, Ib, Ic are used to represent different arms.

### Inactive Controls: Limitations and Benefits

Exercise intervention RCTs included in the analysis most frequently adopted “usual care” as the control condition design (n = 25, 78.1%). Nevertheless, the contents of the usual care activities can vary among studies (Edmond et al., 2019; Lindquist et al., 2007; Thompson & Schoenfeld, 2007). For instance, in a study of a 12-week exercise intervention for prostate cancer survivors, participants in the control arm were not referred to the exercise program. Instead, they received usual care which did not involve any physical activity recommendations (Craike et al., 2018). In another study testing the effects of a 16-week home-based exercise program among acute lymphoblastic leukemia survivors, participants in the usual care control arm received a “physical activity and usual care brochure” identical to that of the intervention group, and were instructed to continue their normal activity levels without restrictions placed on exercising throughout the study period (Manchola-Gonzalez et al., 2020). Lastly, in a study of an eight-week exercise program for lung cancer survivors, the researchers described that the usual care control arm receives general health education material, without specifying the contents of the material (Dhillon et al., 2017). This lack of standardization to quantify the contents of activities in the control condition is considered a key limitation of inactive control designs (Kinser & Robins, 2013; Street & Luoma, 2002; Thompson & Schoenfeld, 2007). As a result, the frequency of participant contacts and the level of attention received among the study arms may vary tremendously (Street & Luoma, 2002).

Using a design that assigns participants to usual care or wait-list control will also render the blinding of participants and research interventionists impossible. Moreover, it is also observed that there is an inadequate reporting, documentation, or oversight of what activities comprise usual care conditions across study settings (Karlsson & Bergmark, 2015). For example, authors may provide limited details regarding the duration of usual care activities or the implementation of standardization of usual care activities. This non-transparency in study design reporting might further conceal the effects of certain biases and non-specific actors that impact the study outcome (Kinser & Robins, 2013; Street & Luoma, 2002; Thompson & Schoenfeld, 2007). The inability to differentiate the treatment effects and nonspecific treatment effects can eventually lead to the inability for researchers to ascertain the intervention effectiveness.

Despite their limitations, inactive control designs are still preferable in many circumstances. Studies adopting inactive control designs such as wait-list and usual care are ideal to control for threats to internal validities including regression to the mean and spontaneous improvement due to the course of illness (Locher et al., 2018). Such designs will be more likely to produce larger effect sizes than a study comparing two active interventions (Kinser & Robins, 2013; Locher et al., 2018). Furthermore, studies using an inactive control group also require smaller sample sizes and fewer resources overall (Williams et al., 2016). Three studies that adopted the wait-list control design in the literature review reported that the use of wait-list control had enhanced study participants recruitment and retention (Chang et al., 2020; Hartman et al., 2018). Wait-list control is often considered an ethical design, given that all participants will eventually receive the treatment intervention. Because the beneficial effects of exercising are well-established and well-known among the general population, this design is particularly appealing and credible to the study participants because they are guaranteed the benefits of the exercise intervention (Hecksteden et al., 2018).

### Active Controls: Limitations and Benefits

In exercise interventions, a variety of attention control designs have been implemented. Among the studies included for analysis in this paper, seven RCTs had an active control condition, and five RCTs employed a multiple-arms design. The component control design was implemented in the study aiming to test the efficacy of weightlifting training to preserve muscle mass among a group of breast cancer survivors. In the study, both the control and intervention groups received a 13-week supervised exercise program. The hypothesized active ingredient, weight training, was added-on to the program only in the intervention group (Brown & Schmitz, 2015).

Furthermore, a different dosage of exercise (i.e., different levels of intensity) could be assigned in the intervention and control arms, respectively. For instance, in a study examining the effectiveness of a 4-week high-intensity exercise training program on cardiovascular health among colorectal cancer survivors, the participants in the attention control arm received a moderate-intensity training program of equal length to that of the experimental group (Devin et al., 2016). Likewise, the attention control arm might be assigned to receive a different modality of activity other than exercise training. For instance, Zhou and colleagues designed an RCT where ovarian cancer survivors in the intervention arm were assigned a 6-month home-based aerobic exercise program, and the attention control arm received weekly phone calls from a research team member, along with a book containing ovarian cancer survivorship–related information (Zhou et al., 2017). In multi-arm exercise intervention RCTs, multiple experimental treatment groups of different exercise dosage (Brown et al., 2018; Kampshoff et al., 2015) or different types of exercise training (Garcia-Soidan et al., 2020; Nouri et al., 2020) are compared against an inactive control condition.

Attention control is an ideal comparison condition because it omits the unique ingredients of the intervention treatment while sharing the common factors across conditions to allow for equal measure and comparison (Aycock et al., 2018). In the aforementioned attention control design, researchers aimed to control for nonspecific factors including the amount of attention, participant expectancy, treatment contact, and social support given to both study arms (Aycock et al., 2018). Attention control, therefore, allows researchers to confer an unambiguous conclusion of the hypothesized unique component of the behavioral intervention (Whitehead, 2004). Furthermore, participants in the attention control arm might benefit from the activities assigned regardless (Aycock et al., 2018; Street & Luoma, 2002). Attention control designs present many advantages over the usual care or no-treatment inactive control designs; nevertheless, they are not without limitations. Unfortunately, little guidance concerning the design standards or the required components in an attention control arm for an exercise intervention has been established in the literature (Hecksteden et al., 2018).

Holding the important active study variables constant across study arms (e.g. intensity and timing of intervention) could be challenging (Pagoto et al., 2013). The amount of attention time participants in the control arm receive might not be equivalent to that received by the participants in the intervention arm. Since RCTs often require considerable human resources, time, infrastructure, and financial support, the resources allowance of a given study might constrain the extent to which the activities in the attention control are set up in parallel to the intervention arm (Lindquist et al., 2007). Researchers must also carefully consider the effects that the activities in the control arm might have on the study outcomes (Aycock et al., 2018; Lindquist et al., 2007). Finally, when the attention control consists of activities with completely different structure, modality, or type as compared with the intervention arm, it is also possible that the attention control activities alter the participant's behavior and through a different mechanism of action, thus becoming an alternative intervention itself. If the control arm shares too many common therapeutic qualities with the intervention arm, it renders the control condition limited in comparability with the intervention treatment (Aycock et al., 2018; Gross, 2005). As a result, it becomes difficult to infer an unambiguous cause-and-effect relationship between the active ingredient and desired outcome specified in the study hypothesis (Kinser & Robins, 2013).

## Discussion: Considerations and Recommendations

Researchers investigating exercise interventions for cancer survivors face numerous challenges in optimal study design, which are predominantly attributed to the multi-component and multifaced nature of such interventions (Hecksteden et al., 2018; Pinto & Floyd, 2007). Based upon the brief review of current literature, it is apparent that there are numerous pitfalls surrounding control condition designs in RCTs of exercise interventions. A flawless RCT design does not exist, and the researcher must consider each option and make careful decisions by weighing the pros and cons when choosing the most appropriate approach in their studies. Researchers should focus on several considerations while designing a control condition in RCTs of exercise interventions targeting cancer survivors. Four principal considerations concerning control condition design, are discussed in this last section of the paper: appropriateness, credibility, appeal, and comparability. Recommendations on how to avoid some major threats to validity and potential biases are also provided along with the discussion.

### Appropriateness

One fundamental principle in control condition design in RCTs is appropriateness. A control condition should be aligned with the overall purpose and objectives of the study, thus enabling the study outcomes to answer the research questions unequivocally (Kinser & Robins, 2013). For instance, when the primary research question is to detect the efficacy of a specific type of exercise training to lessen anxiety among cancer survivors, the intervention arm should be compared to a control condition adopting a different activity while holding the same level of attention and social contact constant (Street & Luoma, 2002). Inactive control such as usual care or waitlist control could also be used to establish the efficacy of the intervention treatment, but the researchers have to take into consideration if the inactive control allows them to adequately isolate the intervention effects from other non-specific effects such as participant expectation. In other words cautious conclusions about the intervention efficacy should be made (Karlsson & Bergmark, 2015).

Alternatively, if the research question concerns the identification of appropriate dosing (e.g., intensity, strength, length, or frequency) of a specific exercise training, the control should be an active control of the same type of exercise training, prescribed at a different dosage. In this case, inactive control conditions would not be appropriate. Finally, an exercise intervention study could aim to examine the efficacy of a specific mechanism of action (e.g., tailored regime vs. traditional exercise regime) or a theory-based variable (e.g., peer support, self-efficacy) on increasing cancer survivors’ level of physical activities (Street & Luoma, 2002; Wolin et al., 2012). For instance, it is common that the exercise intervention consists of training regimes that are personalized to address individual survivors’ physical abilities and specialized needs at the post-treatment transitioning period (Hecksteden et al., 2018; Slade et al., 2016). In this context, active control or wait-list control is the appropriate choice. For ethical reasons, active control or wait-list control are also superior to usual care in cancer survivorship research, considering that the benefits of exercising are well recognized. A no-treatment or usual care control denies the opportunities for the participants to receive a potentially beneficial intervention based on random chances, which might be regarded as ethically problematic (Elliott & Brown, 2002; Street & Luoma, 2002).

### Credibility

The extent to which the study participants perceive the intervention as credible can affect their response to the intervention treatments (Street & Luoma, 2002). Ideally, the control condition and the intervention treatment in an RCT should be equally **credible** in terms of participant perceptions and expectations. Researchers can enhance the perceived credibility of the trial by ensuring that the control conditions contain as many common elements as the intervention treatment as possible, such as equivalent format, structure, attention, expectancy, social contact, and timing of the activities, all of which have the potential to generate the placebo effect (Lindquist et al., 2007). Having the intervention and control activities of parallel format and structure can ensure that the level of engagement among study participants are equivalent. To illustrate, the activities in both intervention and control arms should have equal numbers of sessions or modules, and the duration of involvement should also equate. Besides, the amount of attention given to the participants, quality of social contact, and timing of activities including follow-up times should not vary between groups (Aycock et al., 2018; Staudacher et al., 2017). For instance, research staff or interventionists, including instructors, counselors, educators, nurses, and data collection staff, should be engaged in an equal amount of interaction with all study participants. In situations where monitoring or follow-up are done remotely, the number of phone calls and time spent with all participants should be equivalent. All of these elements have the potential to generate expectations and relationships that influence study adherence, motivation, study completion, and self-reported outcomes by the participants (Locher et al., 2018; Street & Luoma, 2002).

### Appeals and Potential for Social Threats

Relatedly, another essential consideration when designing the control condition in behavioral intervention RCTs is the participant's perceived overall appeal of the study. Both **participant recruitment and retention** could be impacted by the selection of the control condition (Lindquist et al., 2007; Schwartz et al., 1997; Street & Luoma, 2002; Whitehead, 2004). Recruiting study subjects to participate in clinical trials requires a thorough understanding of the basic structure and design of the study, which includes a random group allocation procedure. Potential participants are aware that they will be randomly assigned into either the intervention arm or the control arm. Participant recruitment could be facilitated if both the intervention treatment and the control conditions are appealing to them. In situations where the control arm receives no treatment or a less appealing alternative treatment or activity, participants in the control arm might experience resentful demoralization or compensatory rivalry, two opposite social threats leading to the same bias in RCTs of behavioral interventions (Horner et al., 2006). Indeed, participants in the control arm can become resentful/discouraged, or retaliatory/competitive for not obtaining the desirable intervention treatment. These issues could be magnified in exercise interventions targeted at the cancer survivor population. It is a fair assumption that cancer survivors are aware of the benefits of initiating health promotion behaviors such as exercising after treatment (Berglund et al., 1997; Pinto & Floyd, 2007). Hence, the exercise treatment assigned to the intervention arm might be favorable to the majority of the potential study participants. In the case of resentful demoralization, participants who eventually get allocated to the control arm might feel neglected and attempt to express their resentment by performing differently or inferiorly, resulting in an overestimation of the treatment efficacy (Berglund et al., 1997; Horner et al., 2006). Alternatively, participants experiencing compensatory rivalry become competitive and attempt to compensate for not receiving the desired treatment by increase efforts and seeking alternative means to achieve the same benefits as the intervention treatment, leading to co-intervention and the underestimation of the intervention efficacy (Berglund et al., 1997; Horner et al., 2006). In summary, the emotional and behavioral responses elicited by resentful demoralization and compensatory rivalry are quite the opposite, yet they both might lead to substantial systematic effects in the outcome of the control arm and threatening the construct validity of the intervention, leading to an ambiguous conclusion of study results, as well as diminishing the generalizability of the findings (Berglund et al., 1997; Schwartz et al., 1997).

Participant retention is also associated with the perceived appeal of the study. Participants who regard the control condition as unappealing could withdraw from the study, resulting in attrition, and their data not being available for the final analyses procedure (Davidson & Kaszniak, 2015; Pagoto et al., 2013; Street & Luoma, 2002). Researchers should adopt strategies to enhance the appeal of the study. Matching the control condition with the potential participants’ needs and interests should be considered (Kinser & Robins, 2013; Lindquist et al., 2007). For instance, in a study investigating the effect of a weight training exercise intervention on cancer survivors’ muscle strength, offering the control arm participants a comparable alternative such as aerobic exercise could be an ideal design, since the ultimate desired outcome of the study is health promotion in the post-treatment period in both cases. Seeking a comparable yet acceptable control condition might be more challenging in the case of a novel intervention study (Street & Luoma, 2002). Given that the effect of the intervention treatment has not been previously established, an alternative control condition design such as a delay-start or wait-list control group would be ideal.

### Comparability

Comparability of the intervention treatment and control condition must be taken into consideration in the study design. Researchers need to warrant that the study outcomes are not attributable to the activities assigned in the control arm through either the same or a different mechanism as the intervention treatment (Aycock et al., 2018; Lindquist et al., 2007; Locher et al., 2018). A fundamental function of using a comparison group in an RCTs is that it provides a comparable condition (i.e., a condition able to control for the nonspecific features such as the natural course of the disease) to that of the intervention, thus allowing researchers to draw a conclusion regarding the efficacy of the hypothesized active ingredient (Aycock et al., 2018; Karlsson & Bergmark, 2015; Whitehead, 2004). Preferably, similar to the premises given to ensuring the credibility of the study, a comparable control condition should have a parallel design to that of the intervention group, with the sole difference being the active feature in the study hypothesis. Nonetheless, given the level of complexity in behavioral intervention studies, the researcher must ensure that the activity in the control condition does not unintentionally skew the study outcome. This issue can be addressed by limiting the possibility of **cross-contamination** between the control and intervention arms (Craig et al., 2008; Edmond et al., 2019). More specifically, the activities assigned in the control arm should not have the same mechanism of action that might affect, or contaminate, the study outcome (Edmond et al., 2019). For instance, the interventionists or intervention arm participants might share the intervention elements to the control arm participants unintentionally during interactions throughout the study. In a study where the control arm participants are assigned activities of the same mechanism of action as the intervention arm (e.g., unsupervised exercise vs. supervised exercise), cross-contamination is highly possible.

Tackling the issue of comparability is challenging in behavioral intervention RCTs, particularly in exercise intervention (Hecksteden et al., 2018). Elements in exercise intervention studies such as format and the nature of engagement could be difficult to mimic. To illustrate, if the intervention involves the use of an individualized, tailored exercise prescription, the alternative activities offered in the control arm probably would not generate the same level of participants’ engagement, social contact, and expectancy. Likewise, it is unrealistic to ask participants in the control arm of an exercise intervention study to stay completely inactive (Hecksteden et al., 2018).

### Recommendation for Future Research

Piloting of an RCT is necessary in order to assess for barriers to participant recruitment and to prevent a high rate of attrition (Feeley & Cossette, 2015; Feeley et al., 2009), which can provide insights concerning uncertainties of control condition design of a study. Any issues attributed to control condition designs detected in the pilot trial should be adequately addressed before proceeding to the phase III efficacy trial. In addition, scientific concerns about methodological rigor in RCTs can be overcome by transparent study reporting. It is recommended that researchers provide detailed reports of study design, recruitment, randomization, as well as rationales for choosing a specific design using the guidelines from the CONSORT Statement and the CERT extension (Karlsson & Bergmark, 2015).

## Conclusion

In conclusion, the state of science in exercise training is flourishing in the field of cancer survivorship research. Yet, many systematic reviews have recognized the need for more sophisticated methodological approaches and more appropriate controlled studies (Segal et al., 2017; Stout et al., 2017; Zhi et al., 2019). The gold standard RCT design involves the implementation of placebo controls, which are unfortunately not applicable in most exercise intervention studies. Researchers also need to pay special attention to the design of RCTs, especially concerning the effects of the various types of control conditions on study and participant outcomes. To further examine the challenges in developing optimal control conditions, current literature reporting RCTs of an exercise intervention targeting the cancer survivor population were reviewed in this present paper. Furthermore, to address the methodological challenges of exercise intervention studies, four key considerations were reviewed, and recommendations to address each consideration were provided. A well-designed RCT could deliver valid conclusions about the efficacy of an intervention and can smooth the transition of evidence into clinical practice. More meticulous control condition designs are a crucial step towards making exercise intervention more readily available to cancer survivors.
